# Early amiodarone pneumonitis: A case report

**DOI:** 10.1002/ccr3.6808

**Published:** 2022-12-27

**Authors:** Abbas Soleimani, Houshang Bavandpour Karvane, Masoud Mortezazadeh, Abbas Mofidi, Seyyed Taher Seyyed Mahmoudi, Mehdi Kashani

**Affiliations:** ^1^ Department of Cardiology, Sina Hospital Tehran University of Medical Sciences Tehran Iran; ^2^ Internal Medicine Department, Sina Hospital Tehran University of Medical Sciences Tehran Iran; ^3^ School of Medicine Iran University of Medical Sciences Tehran Iran; ^4^ School of Medicine Tabriz University of Medical Sciences Tabriz Iran; ^5^ School of Medicine Tehran University of Medical Sciences Tehran Iran

**Keywords:** acute lung injury, amiodarone, pneumonitis

## Abstract

Consider amiodarone pneumonitis as an important differential diagnosis of ARDS, especially in clinically ill patients who recently received Intravenous amiodarone.

## Graphical abstract

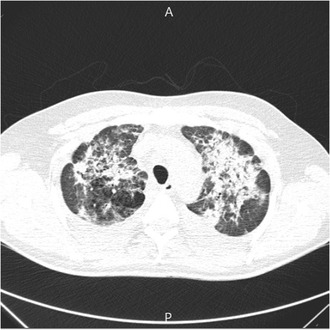

## 1 INTRODUCTION

Amiodarone, a class III antiarrhythmic drug is an iodinated benzofuran derivate used as a potent agent for the prevention and treatment of life‐threatening ventricular and supraventricular arrhythmias.^1^

Its iodine‐containing compound gives amiodarone the tendency to build up in various organs, such as the lungs. Although many adverse reactions and side effects of amiodarone have been reported, pulmonary toxicity and pneumonitis could perhaps be one of the critical adverse effects.^2^

Amiodarone is metabolized in the liver by cytochrome P‐450 enzyme and is then excreted through the biliary system and is not dialyzable in case of overdose.Risk factors for amiodarone pulmonary toxicity consist of the male gender, age, preexisting pulmonary diseases, high oxygen demand conditions, thoracic surgery, and pulmonary angiography.

The most common forms of amiodarone pulmonary toxicity are interstitial pneumonitis and organizing pneumonia, but it can also present itself as a lung mass or a consolidation.

Amiodarone pneumonitis usually manifests itself with non‐specific respiratory symptoms such as cough, shortness of breath, myalgia, and fever.^3^

Due to these indefinite presentations, amiodarone pneumonitis is mostly diagnosed after excluding other differential diagnoses.

Amiodarone pulmonary toxicity is mostly delayed and occurs in higher accumulative dosages, in this case despite most reported cases, we report challenging early amiodarone‐associated pneumonitis.^4^

## 2 CASE PRESENTATION

A 50‐year‐old heavy ex‐smoker man with a past medical history of hyperlipidemia and hypertension was brought to the emergency department because of recent complaints of chest pain, sweating, nausea, and progressive dyspnea.

The initial physical examination showed a body temperature of 37.5°C, blood pressure of 96/70 mm Hg, pulse rate of 95 beats/min, respiratory rate of 26 breaths/min, and oxygen saturation of 90% while the patient was breathing without supplemental oxygen.

He also had bilateral crackles in auscultation, especially in the right lung, Other physical examination findings were unremarkable.

**FIGURE 1:**
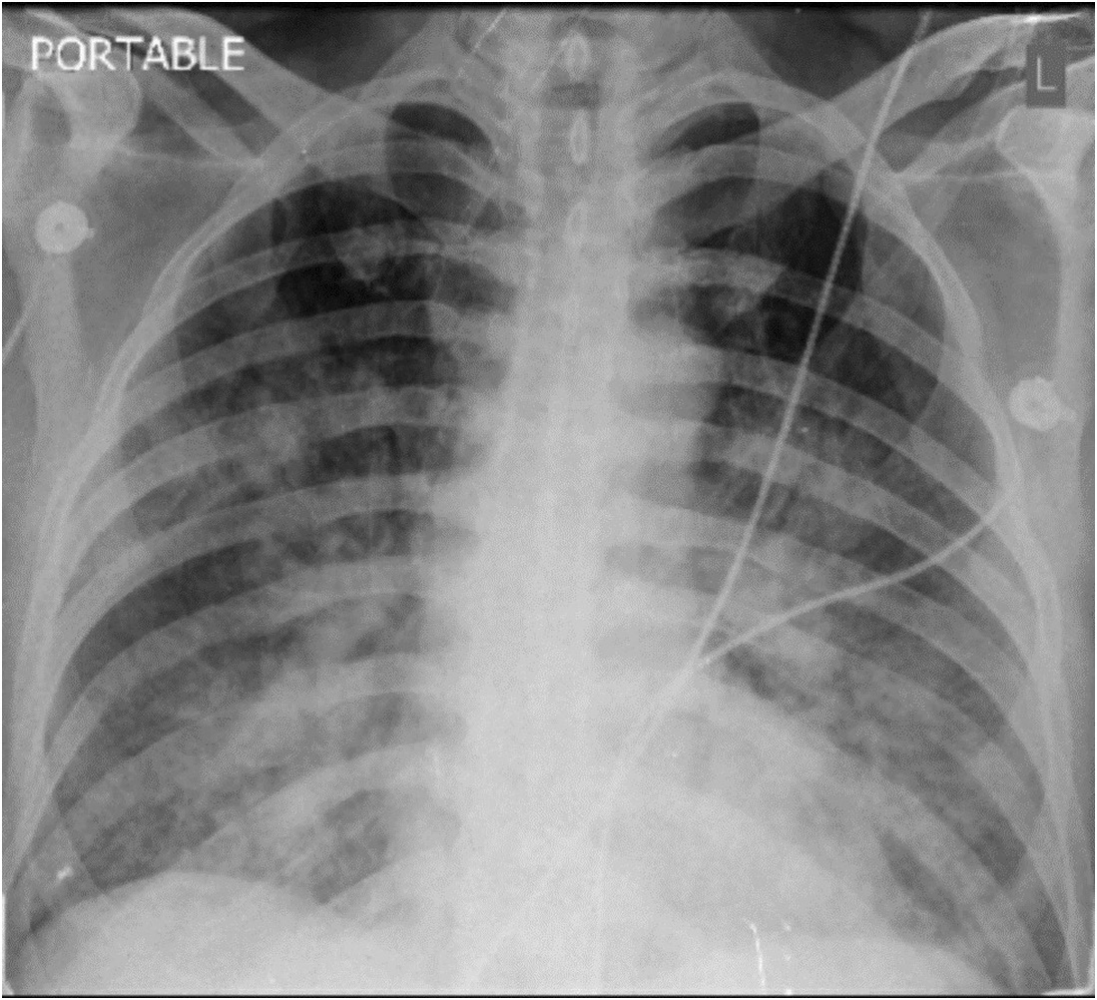
CXR demonstrate the appearance of peri‐hilar consolidation in the context of pulmonary edema.

Immediately ECG and portable chest x‐ray were obtained which demonstrate ST segment elevation in precordial leads and bat wing pattern in CXR in favor of pulmonary edema (Figure 1).

In less than 15 min, the patient was sent to a catheterization laboratory and angiographic imaging revealed total occlusion of the left main artery.

Primary percutaneous catheter intervention and stenting on LAD and plain old balloon angioplasty (POBA) on the ramus intermedius artery were performed.

During the intervention he received, 150 mg amiodarone IV bolus in 10 min, 200 mg bolus of furosemide, heparin 12,500 U, ondansetron 4 mg, epinephrine infusion 2 mcg/kg/min.

After the patient was sent to the critical care unit, he experienced recurrent ventricular tachycardia episodes which 360 mg amiodarone over the next 6 h, then 540 mg over the remaining 18 h administered.

Other medications such as IV furosemide 40 mg twice a day, dobutamine infusion (5 mg/h and 5 mcg/kg/min respectively) and oral spironolactone (25 mg twice a day), aspirin (80 mg daily) Clopidogrel (75 mg daily), and ivabradine (5 mg daily) were administered.

The next day patient's dyspnea gradually improved and amiodarone (200 mg BID) and furosemide (40 mg BID) changed to the oral form.

An echocardiography was performed and an ejection fraction of 20%, pulmonary artery pressure: 45 mm Hg, severe left ventricular hypertrophy, diastolic dysfunction of grade 3, normal right ventricle size, antro‐septal, and apex akinesia, without significant valvular abnormality, and pericardial effusion were reported.

Patient's general condition was improving however on the 7th day of admission the patient developed worsening dyspnea, fever, and nonproductive cough.

Routine biochemistry, CBC diff, CKMb, blood culture, procalcitonin, autoimmune profiles, ESR, CRP, COVID‐19 PCR, and sputum culture samples were obtained and empirical antibiotics (levofloxacin 750 mg daily, ceftriaxone 1 g BID) administered and furosemide dosage was increased.

The ECG did not reveal any new changes.

**FIGURE 2:**
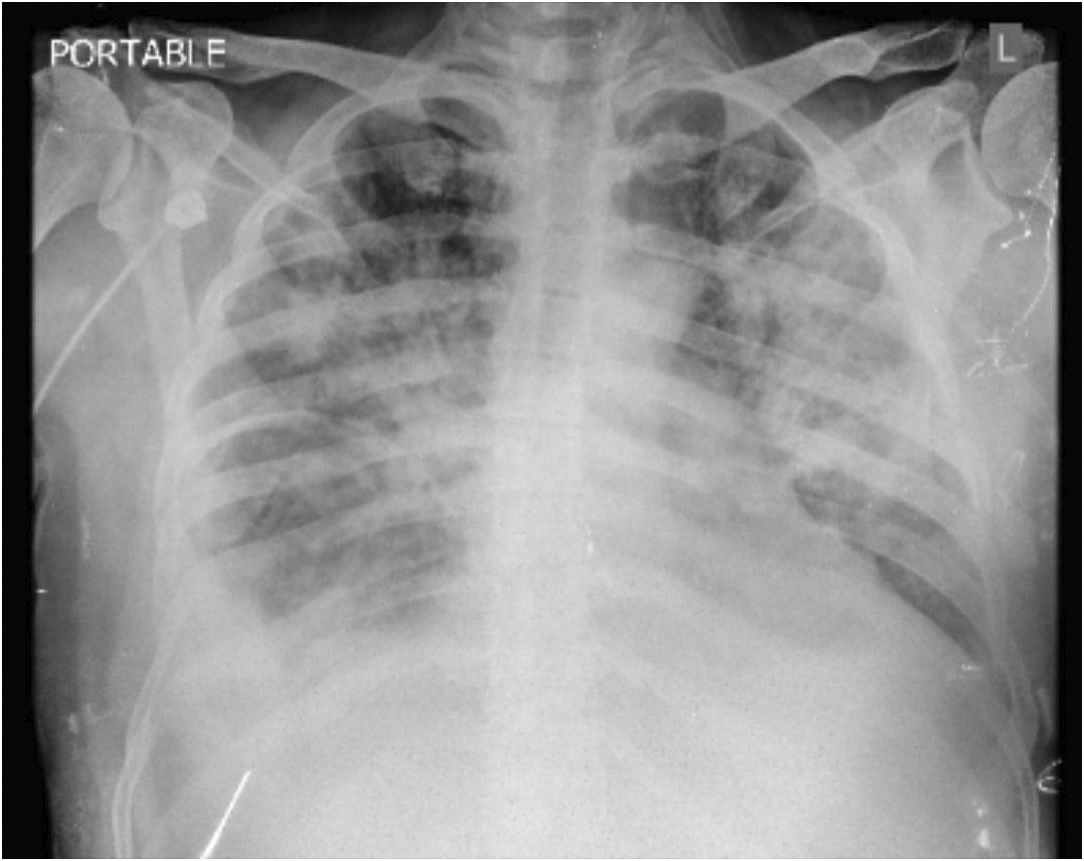
Diffuse bilateral alveolar infiltrates and right pleural effusion.

Follow‐up chest radiography was requested which demonstrated bilateral interstitial infiltrations (Figure 2).

Laboratory results showed WBC 14.8 K/μl, Hgb 11 g/dl, PLT count 250,000 cell/mm^3^, MCV 90.6 fl, neutrophil 70.9%, lymphocyte 27.2%, eosinophil: 1%, CRP:42.7 mg/L, urea 46 mg/dl, Cr 1.4 mg/dl, blood and sputum culture: negative, procalcitonin: negative, CKMb: negative, COVID‐19 PCR (two times with the interval of 48 h): negative, ANA: negative, complements: normal.

**FIGURE 3:**
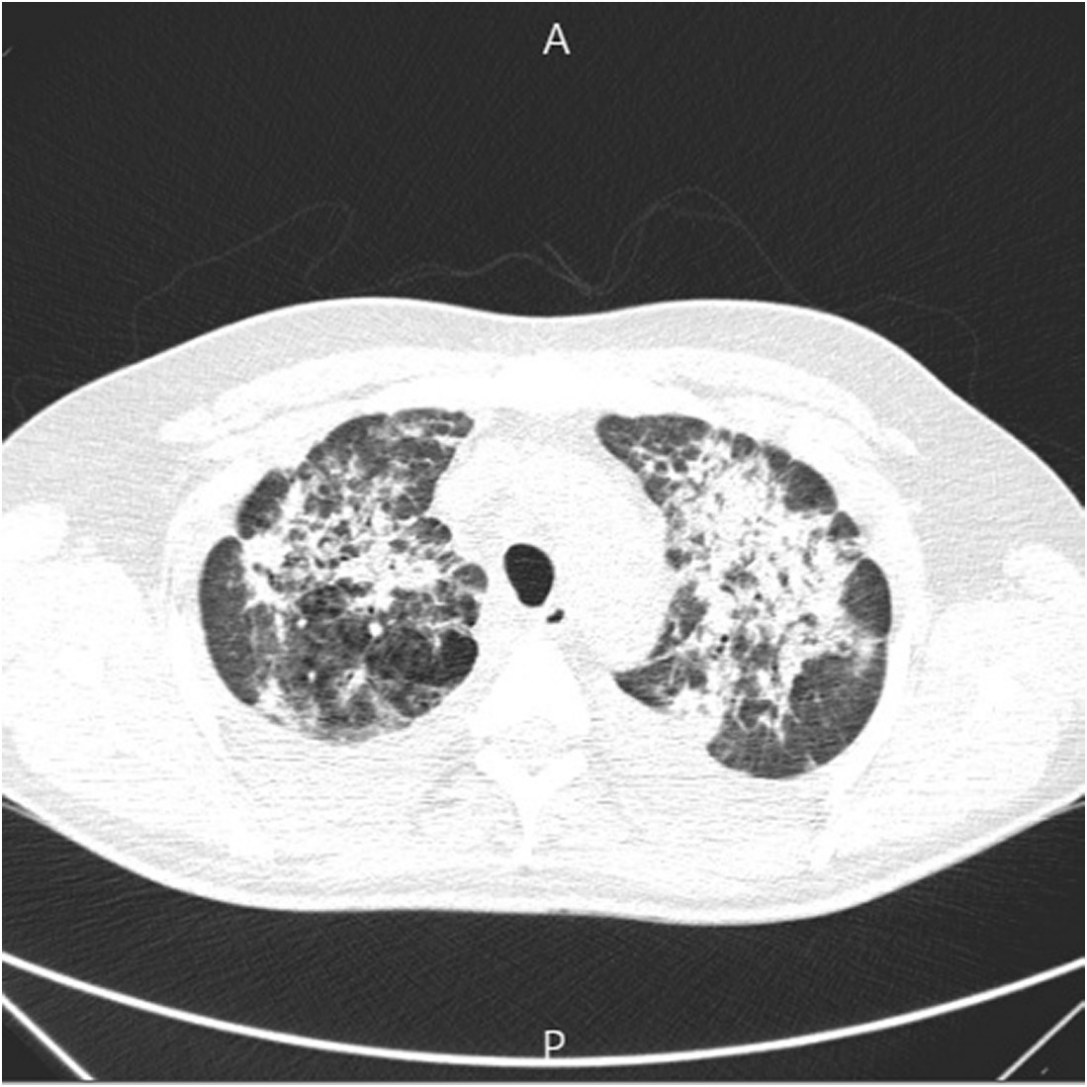
Bilateral diffuse consolidations and ground glass opacities.

Spiral chest computed tomography was requested which showed bilateral diffuse consolidations and ground glass opacities (Figure 3).

First of all with the diagnosis of pulmonary edema and diuretic resistance high dose of furosemide in combination with indapamide, hydrochlorothiazide, and metolazone was subsequently administered but no improvement was seen.

The echocardiography after the worsening of the symptoms demonstrated no change in left ventricle dysfunction, and ejection fraction, but an increase in pulmonary arterial pressure (PAP) to 60 mm Hg which after ruling out the pulmonary emboli the PAP increase was compatible with lung parenchymal involvement.

Due to the worsening of symptoms despite high dose diuretic, broad‐spectrum antibiotic treatment, and negative laboratory results for both viral and bacterial pneumonia and the CT scan consistent with interstitial pneumonitis, amiodarone‐related interstitial pneumonitis was highly suspected and the patient candidate for bronchoalveolar lavage (BAL) and dexamethasone 8 mg IV daily started.

Despite the withdrawal of amiodarone his condition rapidly deteriorated within 3 days and suffered respiratory and kidney failure and cardiac arrest during hemodialysis. He underwent cardiorespiratory resuscitation for over 45 min but could not be resuscitated and unfortunately died.

## 3 DISCUSSION

Amiodarone is one of the most commonly prescribed medications for the treatment of life‐threatening ventricular arrhythmia.

Treatment with amiodarone has various known neurologic, ophthalmic, bone marrow, cutaneous, thyroid, hepatic, and pulmonary side effects.

Pulmonary toxicity is among the most serious adverse effects of amiodarone.

Interstitial pneumonitis, eosinophilic pneumonia, organizing pneumonia, acute respiratory distress syndrome, diffuse alveolar hemorrhage, lung mass or consolidation, and rarely pleural effusion can occur in amiodarone toxicity.^5^

Among these various types of pulmonary adverse effects, interstitial pneumonitis is the most common presentation after two or more months of treatment, particularly in individuals over 60 years old with preexisting lung disease and whose daily amiodarone dosage exceeds 400 mg could occur.

Most of these effects occur due to the accumulation of amiodarone metabolite so it rarely manifests in the early stage of administration like in our case.

Interstitial pneumonitis due to amiodarone toxicity is characterized by the sudden onset of nonproductive cough, dyspnea, and less likely malaise and fever could be seen. Our case also presented with abrupt onset of mentioned symptoms which could not be explained by pneumonia or pulmonary edema.

Other rare microorganisms such as fungal infection in immune‐competent individuals were also unexpected.

A negative autoimmune panel was also more compatible with amiodarone pneumonitis diagnosis.

When the clinical diagnosis of amiodarone‐induced interstitial pneumonitis is questionable, flexible bronchoscopy with (BAL) is done. This procedure is more beneficial in excluding other differential diagnoses.

Despite chronic amiodarone pneumonitis which lipoid cells are more likely to be seen in pathology in acute amiodarone pneumonitis BAL investigation, alveolar hemorrhage is more probable to be detected.^6^

So, we can conclude that the absence of lipoid macrophages in BAL is not supported by excluding the diagnosis of amiodarone pneumonitis and clinical and radiologic features such as abrupt worsening dyspnea and cough, administering a high dosage of amiodarone, exclusion of lung infection and hypersensitivity pneumonitis, and new ground glass on chest radiography are key features for diagnosis of early amiodarone pneumonitis.

Even if our patient had the chance for the BAL, it is not the gold standard for diagnosing amiodarone pneumonitis and as mentioned in previous studies only 50% of patients with amiodarone lung injury manifest with lipoid macrophages in BAL, on the other hand these lipoid cells could be seen in non‐toxic patients who are taking amiodarone.^7^

Previous studies also mentioned that the route of administration might be another factor contributing to acute amiodarone pneumonitis like our reported case, patients who received amiodarone 150 mg IV in less than an hour, followed by exceeding dose of 1000 mg IV in a day, amiodarone metabolites can accumulate more rapidly in lungs.^8^

On the other hand, some studies believe that acute oxidant lung injury also plays a major role in amiodarone lung injury in the acute phase.^9^

Treatment of amiodarone pneumonitis consists primarily of cessation of amiodarone and, in severe cases, initiating systemic glucocorticoids (prednisone 40–60 mg per day or methylprednisolone 500–1000 mg/day intravenously in severe cases) is recommended.^10^

It is reasonable not to administer antiarrhythmic with similar structure in amiodarone lung injuries such as dronedarone, and a personalized approach for each patient is recommended especially in the elderly patient with underlying lung diseases such as previous severe COVID‐19 which lead to permanent lung injury.^11^, ^12^, ^13^, ^14^, ^15^

Acute amiodarone‐related interstitial pneumonitis is a rare and more aggressive form of the disease, most often occurring in critically ill and post‐surgery patients. Acute respiratory distress syndrome (ARDS) and alveolar hemorrhage are the most common pattern of amiodarone pneumonitis, our case also encountered ARDS despite corticosteroid treatment.^16^, ^17^

## 4 CONCLUSION

In brief, our challenging case illustrates the difficulties in the diagnosis of acute amiodarone pneumonitis which should be considered as a differential diagnosis of ARDS causes especially in patients whose other related diagnoses are excluded and who had a history of receiving a high dosage of intravenous amiodarone.

## AUTHOR CONTRIBUTIONS

**Abbas Soleimani:** Writing – original draft; writing – review and editing. **Houshang Bavandpour Karvane:** Writing – original draft. **Masoud Mortezazadeh:** Supervision; writing – original draft; writing – review and editing. **Abbas Mofidi:** Writing – original draft. **Seyyed Taher Seyyed Mahmoudi:** Writing – original draft. **Mehdi Kashani:** Writing – original draft; writing – review and editing.

## FUNDING INFORMATION

None declared.

## CONFLICT OF INTEREST

The authors declare that there is no conflict of interest to declare.

## ETHICAL APPROVAL

In this study, no additional costs and procedures were imposed on the patient's family members. We reported the retrograde standard treatment process of the patient. We maintained the patient's privacy.

## CONSENT

The patient's family has consented to the participation of this case report. The patient's next of kin (according to our hospital policy, the eldest male child) has consented to the publication of this case report.

## Data Availability

The data that support the findings of this study are available from the corresponding author [MM], upon reasonable request.
